# Novel Additive Manufactured Multielectrode Electrochemical Cell with Honeycomb Inspired Design for the Detection of Methyl Parathion in Honey Samples

**DOI:** 10.1021/acsmeasuresciau.3c00003

**Published:** 2023-04-06

**Authors:** Bruno C. Janegitz, Robert D. Crapnell, Paulo Roberto de Oliveira, Cristiane Kalinke, Matthew J. Whittingham, Alejandro Garcia-Miranda Ferrari, Craig E. Banks

**Affiliations:** † Department of Nature Sciences, Mathematics, and Education, Federal University of São Carlos, 13600-970 Araras, São Paulo, Brazil; ‡ Faculty of Science and Engineering, Manchester Metropolitan University, Manchester M1 5GD, United Kingdom; § Institute of Chemistry, University of Campinas (Unicamp), 13083-859 Campinas, São Paulo, Brazil

**Keywords:** Additive Manufacturing, 3D Printing, Multielectrode, Fused Filament Fabrication, Fused Deposition Modeling, Electrochemistry, Food Samples

## Abstract

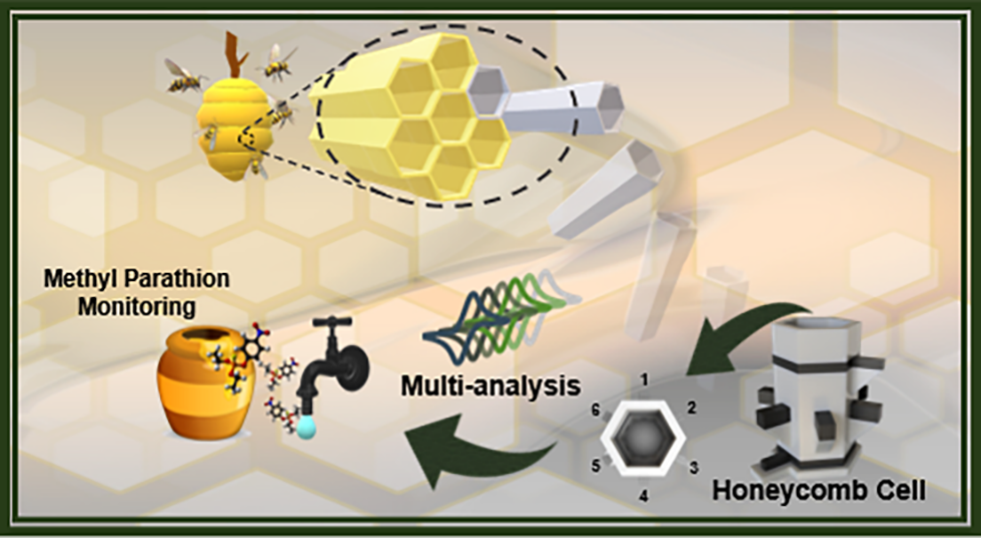

The development and increase in the number of crops recently have led to the requirement for greater efficiency in world food production and greater consumption of pesticides. In this context, the widespread use of pesticides has affected the decrease in the population of pollinating insects and has caused food contamination. Therefore, simple, low-cost, and quick analytical methods can be interesting alternatives for checking the quality of foods such as honey. In this work, we propose a new additively manufactured (3D-printed) device inspired by a honeycomb cell, with 6 working electrodes for the direct electrochemical analysis of methyl parathion by reduction process monitoring in food and environmental samples. Under optimized parameters, the proposed sensor presented a linear range between 0.85 and 19.6 μmol L^–1^, with a limit of detection of 0.20 μmol L^–1^. The sensors were successfully applied in honey and tap water samples by using the standard addition method. The proposed honeycomb cell made of polylactic acid and commercial conductive filament is easy to construct, and there is no need for chemical treatments to be used. These devices based on 6 working electrodes array are versatile platforms for rapid, highly repeatable analysis in food and environment, capable of performing detection in low concentrations.

## 1 Introduction

The increase in the number of crops in recent years has led to needing greater efficiency in world food production.^1^ However, as a consequence, there has been a need to increase the application of pesticides.^2^ The use of pesticides can lead to human consumption of these substances directly or indirectly. Another issue is the dispersion of these substances beyond the intended application site by air, soil, or carried by water sources. An example of this is the contamination of water and honey, caused by contact with toxic substances used in monocultures.^3^ This harmful contact can be extended not only to the human population that feeds on these products but also to small animals present in this environment that make use of these affected plants. These substances can also translocate between plants^4^ such as angiosperm plants, which produce pollen. Simultaneously, there is a decline in pollinators across the globe, a phenomenon attributed to several factors such as the expansion of agricultural practices, fragmentation of habitats, and use of pesticides, with the latter being recognized as the main cause.^3^ For the maintenance and improvement of ecosystems, pollinating agents are considered crucial, and among them, bees have enormous prominence.

Bees are strongly affected by this contamination, which can lead to death or behavioral changes in these individuals.^5^ The individual contamination of a bee can be amplified when it transports the contaminant into the hive, contaminating the entire colony and the honey they produce.^2^ The evaluation and determination of toxic species in bee honey can be a good strategy for estimating the degree of contamination of these insects. Bee products such as bee pollen, honey, and royal jelly are popular agricultural products around the world. These exhibit interesting bioactivities such as antimicrobial, anti-inflammatory, and antioxidant actions.^4^ It is important to contextually analyze the presence of pesticides in honey samples, which can endanger the reproduction of this insect as well as the health of the human beings who consume this product. In addition, the detection of pesticides can provide data for the risk assessment of different bee species more consistently.

The broad use of organophosphate pesticides for pest control has caused serious concerns regarding human health, food safety, and environmental protection.^6,7^ Methyl parathion (MP) is one of these organophosphate insecticides—with the molecular formula C_8_H_10_NO_5_PS—which is used on a large scale in several countries,^8^ commonly used in agricultural fields to prevent the attack of insects.^6^ As a result, it is found in water, food, and soil, which is potentially toxic to human health.^9^ MP can be detected using analytical techniques such as chromatography,^10^ immunoassays,^11^ molecular imprinting,^12^ and mass spectrometry.^13^ In this sense, new procedures as alternatives for the detection of this agrochemical are very important for food and environmental analysis to reduce costs and allow for on-site measurements.

Additive manufacturing technology is recent and has great potential for building objects with greater practicality in a shorter time.^14^ The advancement of this technology has enabled an improvement in both print resolution and a reduction in production costs.^14,15^ Its efficiency—when compared to traditional prototyping methods—stands out, particularly due to the ability to produce different types and designs of devices on the same 3D printer without retooling, which characterizes a great production potential and versatility.^16^

With the development of the technique, the construction of complex structures became possible, since this technique allows the easy and fast production of precise parts. In contrast, alternative techniques such as injection molding and embossing have emerged to compete for market demand.^17^ Also, additive manufacturing is advantageous due to the relatively low cost of polymers, further reducing the cost and also offering the advantage of recyclable materials, such as polylactic acid (PLA).^18^

Additive manufacturing technologies made it possible to reduce (or eliminate) the transport of samples from the collection site, enabling quick local analysis. Therefore, an additive manufacturing collection system can be an alternative to in situ analysis, which makes this an even more interesting avenue of research.^19^

Additive manufacturing provided a breakthrough in the area of materials and currently symbolizes the potential to transform the way society manufactures its goods.^20^ Analytical chemistry and electrochemistry have recently benefited from 3D-printing technology.^21,22^ The freedom of design made possible by additive manufacturing opens up many possibilities for researchers to create new materials and electrochemical detection devices to be produced in batches, with the desired geometry with zero waste generation and relatively low cost.^23^ The literature has demonstrated the use of commercially available desktop 3D printers to manufacture functional and low-cost objects.^24^ This approach has also been explored for electrochemical and electroanalytical applications. The extruded filament is deposited layer by layer using an extruder mounted on a Cartesian motion system, consisting of a *X*/*Z*-axis gantry and an *Y*-axis moving build plate, which allows the creation of versatile three-dimensional objects with complex shapes and accurate dimensions.^25,26^ The use of commercial conductive filaments is attractive due to the low cost, design versatility (shape and size), and the possibility of rapid decentralized production of conductive or semiconductor substrates for different electrochemical applications.^27,28^

Although there is a growing trend in the construction of electrochemical cells using 3D printing technology, most of these works aim at segmented construction of the electrochemical cell and electrode arrangement.^29,30^ Hence, the present work aims to develop an entire additive manufacturing electrochemical cell with 6 working multielectrodes in the same device for the detection of methyl parathion in bee honey and tap water samples. In this sense, this is a promising analytical tool to monitor honey quality in situ.

## 2 Experimental Section

### 2.1 Materials and Samples

All chemicals used were of analytical grade and used without purification. Sodium phosphate monobasic (99%) and dibasic (99%), acquired from Merck, were used for the preparations of phosphate buffer solutions. Hexaamineruthenium(III) chloride (RuHex, 98%) was purchased from Merck (Gillingham, UK). Methyl parathion was obtained from Sigma. The commercial conductive PLA/carbon black filament (ProtoPasta, Vancouver, Canada) was purchased from Farnell (Leeds, UK). Commercial PLA filaments were acquired from Prusament, CZ. Real samples of honey (Clear Honey, Tesco) were purchased from a local store. Tap water samples were obtained from a tap of the laboratory located in the John Dalton Building at Manchester Metropolitan University (GPS orientation: 53.472127817377185, −2.2392556202361757). All solutions were prepared with deionized water with a resistivity not less than 18.2 MΩ·cm from a Milli-Q Integral 3 (Merck Millipore, UK). The honey samples were prepared by simple dissolution of 1.0 g of honey in 10 mL of 0.10 mol L^–1^ phosphate buffer (pH 5.1) (1:10 w/v). For homogenization, the samples were stirred for 5 min using a vortex at room temperature. Afterward, the samples were enriched with MP, which was detected by using the proposed system. Also, the tap water samples were prepared by simple dilution in phosphate buffer, which were subsequently spiked with MP for further electrochemical MP detection.

### 2.2 Apparatus

A Metrohm Autolab M204 6 multichannel potentiostat/galvanostat was used to obtain the electrochemical measurements, which were recorded sequentially. For the physicochemical characterization, scanning electron microscopy (SEM) measurements were recorded using a Thermo Fisher Scientific model Prisma E to acquire SEM images with an average chamber and gun vacuum of 1.3 × 10^–5^ and 1 × 10^–9^ mbar, respectively. Samples were mounted on the aluminum SEM pin stubs (40 mm diameter). For the contact angle analysis, a homemade dropometer was used. For the acquisition of images and the angle determination, ImageJ software and a snake-based approach were used, respectively.^31^ For the electrochemical impedance spectroscopy (EIS), 1.0 mmol L^–1^ ferrocenemethanol (FcMeOH) in 0.1 mol L^–1^ KCl was used as the redox probe.

### 2.3 Additive Manufacturing

**Figure 1:**
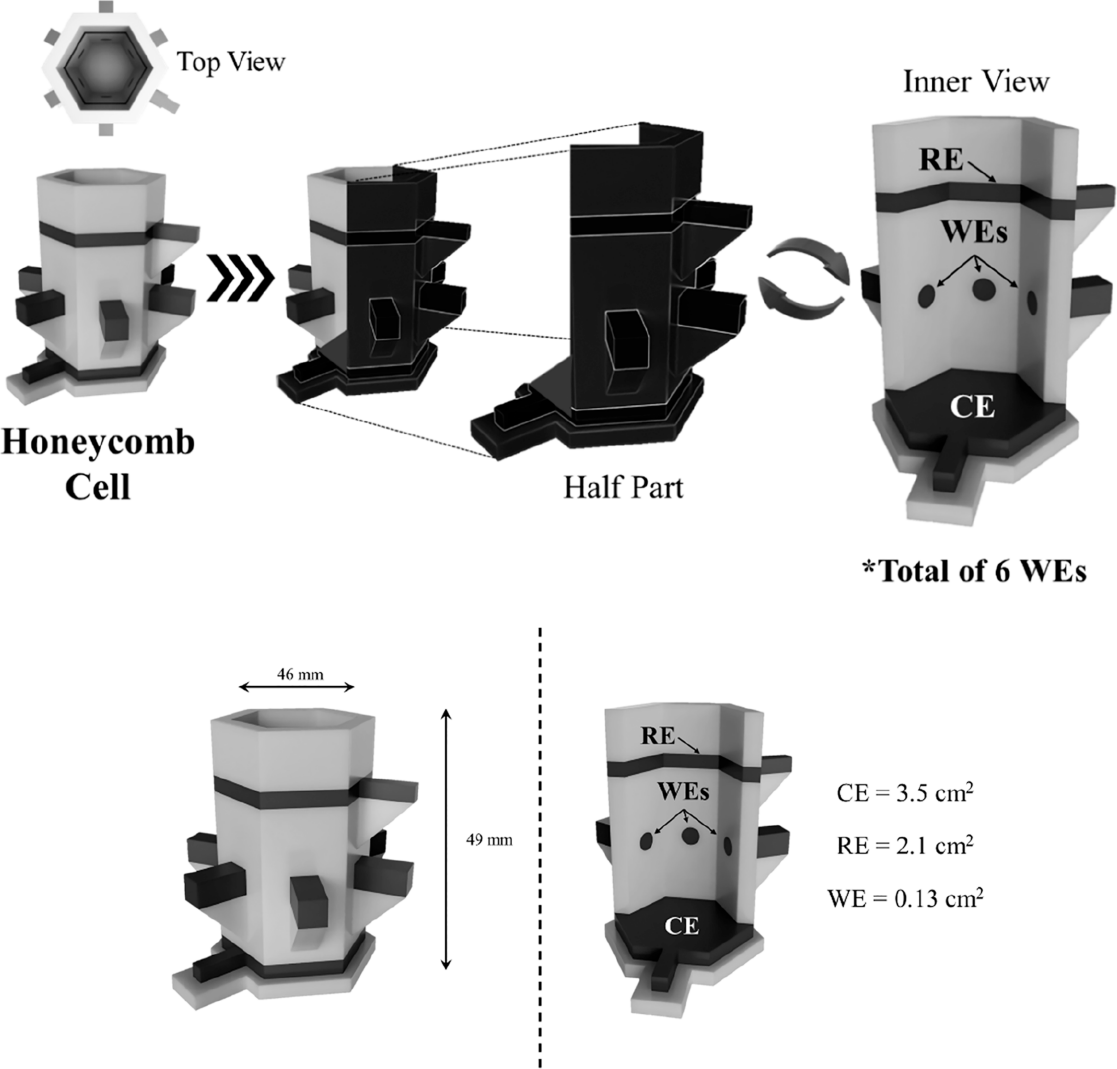
Representative figures of the additively manufactured honeycomb system, with 6 working, reference, and auxiliary electrodes, including the dimensions.

The additively manufactured (AM) electrochemical platform was produced using fused filament fabrication (FFF) on a Raise3D E2 3D printer (Raise3D, California, United States). All designs and .3MF files were produced using Autodesk Fusion 360 and then sliced and converted to G-code files using the open-source software ideaMaker 4.0.1 (Raise3D, California, United States). The .3MF files are available for download from the Supporting Information. The design and dimensions of the cell are shown in Figure 1, including the electrodes and cell sizes. Also, the real images are provided in Figure S1 in the Supporting Information. Additionally, a video taken throughout the printing process is available in the Supporting Information. The electrochemical cells were all printed using the Raise3D E2 independent dual extruder (IDEX) printer with nonconductive Prusament Galaxy Silver PLA filament (Prusa Research, Prague, Czech Republic), printed on the left nozzle (0.4 mm) at a set temperature of 205 °C. For the 6 working electrodes, counter and reference electrode construction, a conductive carbon black/PLA filament (Protopasta, Vancouver, Canada) was printed on the right nozzle (0.4 mm) at a set temperature of 205 °C, which the same material used as the electrical contact of all electrodes. The printing bed temperature was set at 55 °C throughout the prints. The cells were printed using a layer height of 0.2 mm, with a gyroid infill of 10% and an infill speed of 40 mm/s for the standard PLA profile and 100% infill and 40 mm/s speed for the CB/PLA. This print had a purge block located close to the cells, as well as a skirt to help prime the nozzle before printing the first layer and between each extruder change. The print for two cells took 6 h and 9 min to complete using a total of 35.7 g of standard PLA and 11.3 g of CB/PLA. The total volume of the cell is 15 mL, of which 12 mL is sufficient to cover the working and reference electrodes (the counter is on the base).

## 3 Results and Discussion

### 3.1 Physicochemical Characterization

**Figure 2:**
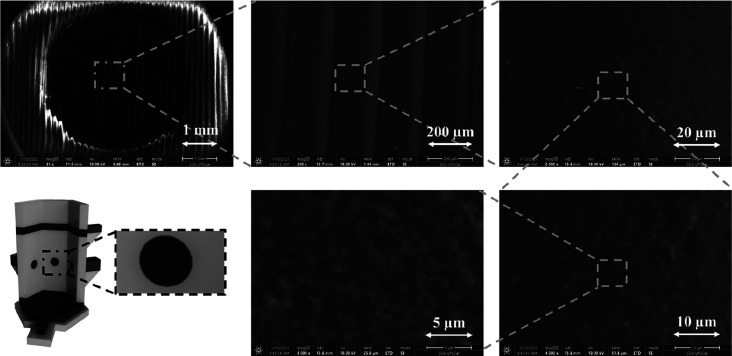
SEM images of the 3D-printed electrode at different magnifications.

Part of the proposed electrochemical system was prepared for scanning electron microscopy and contact angle measurements. For this purpose, one piece of 2.0 cm × 1.0 cm was printed with PLA and carbon black/PLA to represent the working electrodes of the honeycomb cell. Figure 2 shows the SEM images with different magnifications, in which it is possible to observe lines related to the nozzle used for the 3D printing. In the zoomed-in images, it is possible to observe dots in heterogeneous distribution, which are related to the carbon black, the conductive material present in the commercial filament. Figure S2 shows the contact angles measured for 3 different conductive working electrodes obtained at the same condition and without any treatment. The water contact angle for all samples exhibited a mean value of 93.5 ± 1.7°, which is attributed to a hydrophobic behavior in water (>90°).^32^

### 3.2 Electrochemical Performance

**Figure 3:**
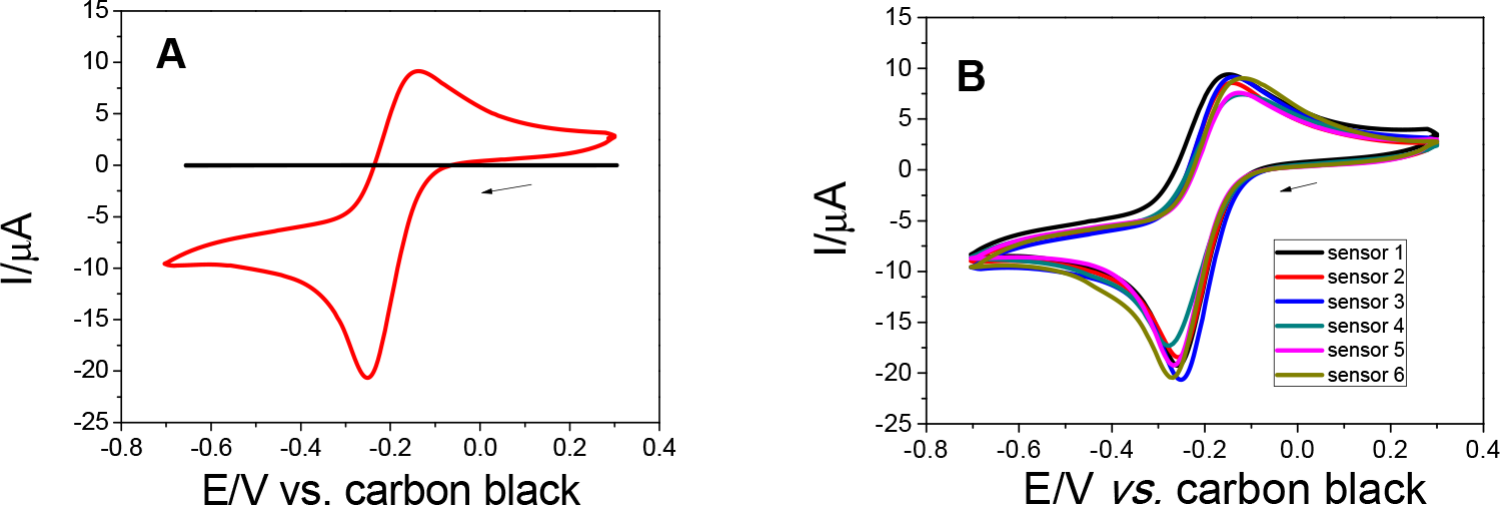
(A) Cyclic voltammograms of an additive manufactured honeycomb system obtained in the absence (black) and in the presence (red) of 1.0 mmol L^–1^ [Ru(NH_3_)_6_]^3+/2+^ in 0.1 mol L^–1^ KCl, at a scan rate of 25 mV s^–1^ using one electrode. (B) Cyclic voltammograms of 6 electrodes (run separately) in the presence of [Ru(NH_3_)_6_]^3+/2+^ at the same conditions.

The main purpose of this work consists of the construction of an all-in-one additively manufactured system in which the 6 working electrodes, auxiliary electrode, and reference electrode are printed in an electrochemical cell in the form of a honeycomb (hexagonal prism). This system could be used for field applications, and the cell itself can be used to collect water/honey for subsequent electrochemical measurement. Some works have shown that electrochemical/chemical treatments or “activations” are necessary to obtain voltammetric signals effectively.^33,34^ However, the intention is to use this additively manufactured system as-is, without postprocessing of the electrodes. In this sense, cyclic voltammetry (CV) measurements were performed on the cell as printed. Figure 3A shows the cyclic voltammogram obtained in the presence of 1.0 mmol L^–1^ RuHex ([Ru(NH_3_)_6_]^3+/2+^) in 0.1 mol L^–1^ KCl using the honeycomb cell at a scan rate of 25 mV s^–1^. We can observe the well-defined anodic peak current with a value of 9.3 μA and the cathodic peak current of −20.6 μA, with the Δ*E*_p_ value of 110 mV. Using the 6 electrodes present in the cell, it is possible to observe the voltammograms with similar profiles and magnitudes (Figure 3B), which provided the relative standard deviation (RSD) value of 8.1%. These values are similar to those of other additive manufactured sensors.^14,35,36^ These types of sensors work well for inner sphere redox probes, as demonstrated in previous papers in the literature.^22,35,37^ The variation in the peak current and the Δ*E*_p_ shows that, in some cases, the AM sensors present electrochemical behavior even without chemical/electrochemical treatments that are necessary to expose the conductive material contained in the (usually) PLA polymer matrix. Also, the non-treated 6 working electrodes have presented good charge transfer resistance (*R*_ct_) values, around 1527 ± 194 Ω (*n* = 3), in comparison to those in other works in the literature.^30,38^ A representative Nyquist plot and the *R*_ct_ values obtained for the 6 electrode arrays can be observed in Figure S3. Therefore, the multielectrode cell proposed proved to be effective, and no surface treatment was necessary to obtain satisfactory responses for the studied electrochemical probe.

**Figure 4:**
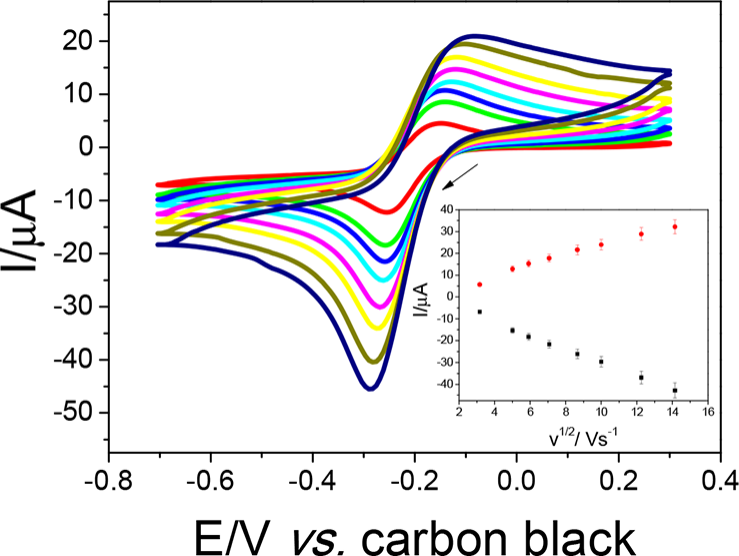
Cyclic voltammograms of the honeycomb system obtained in the presence of 1.0 mmol L^–1^ [Ru(NH3)_6_]^3+/2+^ in 0.1 mol L^–1^ KCl, at scan rate range of 10, 25, 35, 50, 75, 100, 150, and 200 mV s^–1^, and *I* × *v*^1/2^ plot (inset).

The electroactive area of the working electrodes was estimated by cyclic voltammetry (Figure 4) based on the Randles–Ševčík equation^39^ using the *I* versus *v*^1/2^ plot (inset Figure 4). In this regard, scan rates of 10, 25, 35, 50, 75, 100, 150, and 200 mV s^–1^ were applied using 1.0 mmol L^–1^ [Ru(NH_3_)_6_]^3+/2+^ as the redox probe, in 0.1 mol L^–1^ KCl solution as the supporting electrolyte. As observed, the peak currents, anodic and cathodic, increased when the scan rate increased. The average electroactive area obtained for the 6 electrodes was 0.004 cm^2^, smaller than that of the geometric area (0.13 cm^2^). This fact can be related to the absence of chemical/electrochemical treatments and thus the reduced surface area of exposed conductive material in the polymer. The heterogeneous electron transfer constant (HET), *k*^0^_obs_, was determined by the Nicholson method^40^ with the collected CV data, in which the obtained value was 2.81 ± 0.01 × 10^–3^ s^–1^.

### 3.3 Determination of Methyl Parathion Using the Proposed System

**Figure 5:**
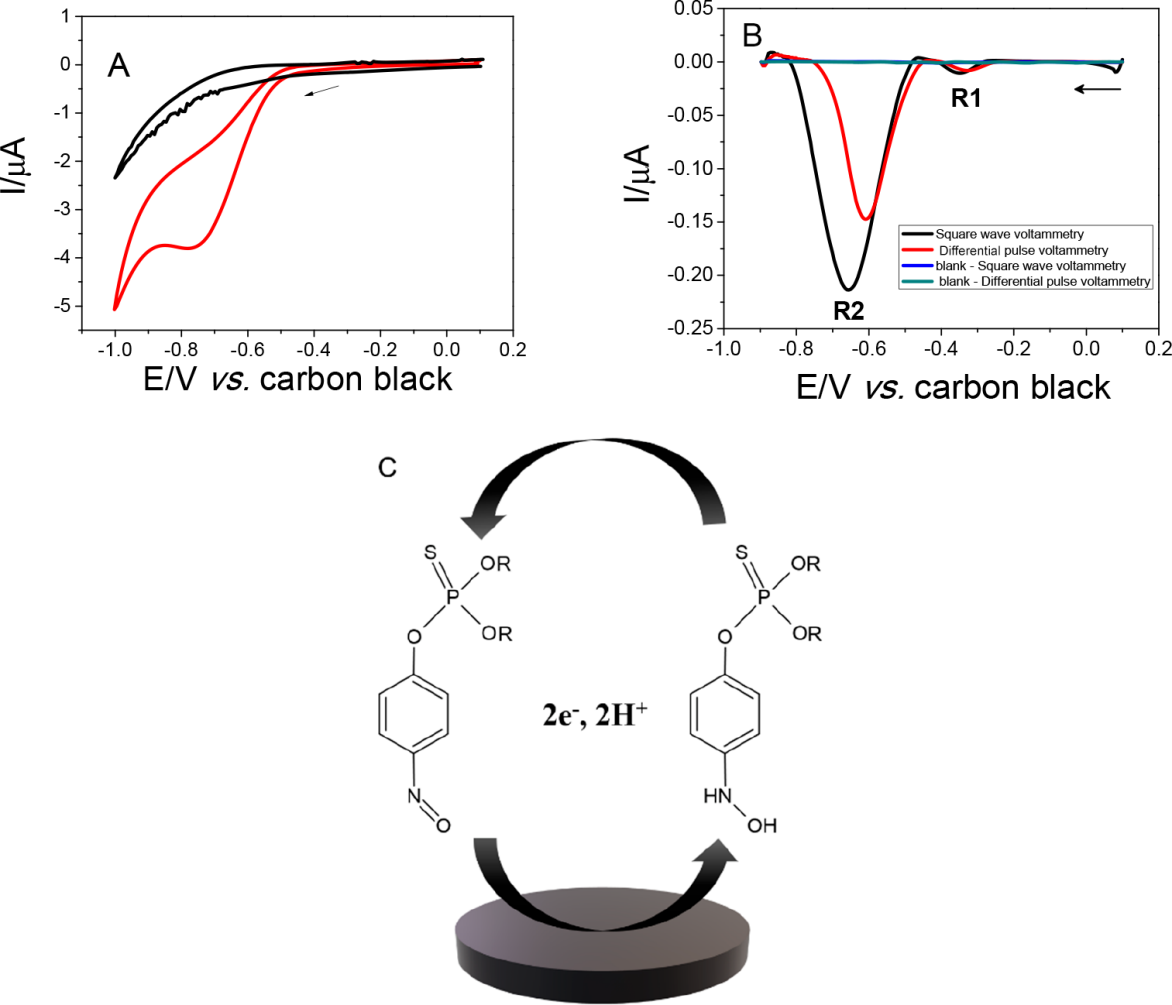
(A) Cyclic voltammograms recorded in the absence (black) and in the presence (red) of 1.0 × 10^–4^ mol L^–1^ MP in 0.1 mol L^–1^ phosphate buffer (pH 7.4) using the additive manufactured honeycomb system; scan rate: 100 mV s^–1^, adsorption time: 900 s. (B) Differential pulse voltammogram (red) and square wave voltammogram recorded in the absence (black) and in the presence of 8.0 × 10^–5^ mol L^–1^ MP in 0.1 mol L^–1^ phosphate buffer (pH 7.4). (B) Use of additive manufactured honeycomb system. Conditions: step potential, 3.0 mV; amplitude, 25 mV; frequency, 30 Hz; adsorption time, 900 s, for square wave voltammetry and scan rate of 30 mV s^–1^ and amplitude of 30 mV for differential pulse voltammetry. (C) Possible mechanisms for MP redox reaction.

First, cyclic voltammetry measurements were carried out to observe the voltammetric behavior of methyl parathion (MP) using the proposed system. The measurements were recorded in the absence and in the presence of 1.0 × 10^–4^ mol L^–1^ MP, in 0.1 mol L^–1^ phosphate buffer (pH 7.4), with an adsorption time of 900 s, in the absence of stirring. In Figure 5A, it is possible to observe a cathodic peak around −0.70 V (vs carbon black) related to the reduction of the nitrogenous group of the MP. Studies were performed with square wave and differential pulse voltammetries to select the best technique for MP quantification. Measurements were carried out using the following parameters: frequency of 30 Hz, square wave amplitude of 25 mV, step potential of 3 mV (square wave voltammetry, SWV) and rate of 30 mV s^–1^, and amplitude of 30 mV (differential pulse voltammetry), in the presence of 8.0 × 10^–5^ mol L^–1^ MP, in 0.1 mol L^–1^ phosphate buffer medium (pH 7.4). In Figure 5B, we observe two peaks, the first reduction at approximately −0.35 V (R1), only detected by the pulsed technique (SWV) due to the capacitive current discount, which does not occur by cyclic voltammetry, and another one at around −0.6 V (R2), attributed to the irreversible reduction process. For the analytical monitoring, the first redox process (R1) was used. This response mechanism presented in Figure 5C can be attributed to the reversible reduction (R1) of hydroxylamine (NHOH) which is converted to nitro (NO) in the MP that involves 2*e*^–^ and 2H^+^, as observed in the literature.^7,41^ To present a high signal for the quantification of the analyte of interest, the square wave voltammetry technique was chosen.

Some parameters were optimized for MP quantification using the proposed system. First, the parameters of voltammetry were studied in the presence of 8.0 × 10^–5^ mol L^–1^ MP in 0.1 mol L^–1^ phosphate buffer medium (pH 7.4). The frequency was studied from 10 to 100 Hz, and the selected value was 100 Hz, which presented a higher analytical signal (Figure S4). Next, the square wave amplitude was studied from 10 to 60 mV, and the value of 40 mV was chosen to present the best resolution in the voltammetric response. Step potential was studied from 1.0 to 10 mV, and the cathode current value increased to 8.0 mV, which was selected for further studies.

The pH of the phosphate buffer was also measured in the range of 5.1 to 7.8 in the presence of 8.0 × 10^–5^ mol L^–1^ MP. There was an inverse relationship between the decreasing buffer pH and the increasing peak current, in which the value of pH 5.1 presented the highest cathodic current and was the value chosen for subsequent experiments. Also, the time of MP absorption was studied from 180 to 500 s in the presence of 8.0 × 10^–5^ mol L^–1^ MP in 0.1 mol L^–1^ phosphate buffer (pH 5.1). The cathodic peak current increased to 420 s and remained constant after that value. Therefore, a time of 420 s was the selected value.

**Figure 6:**
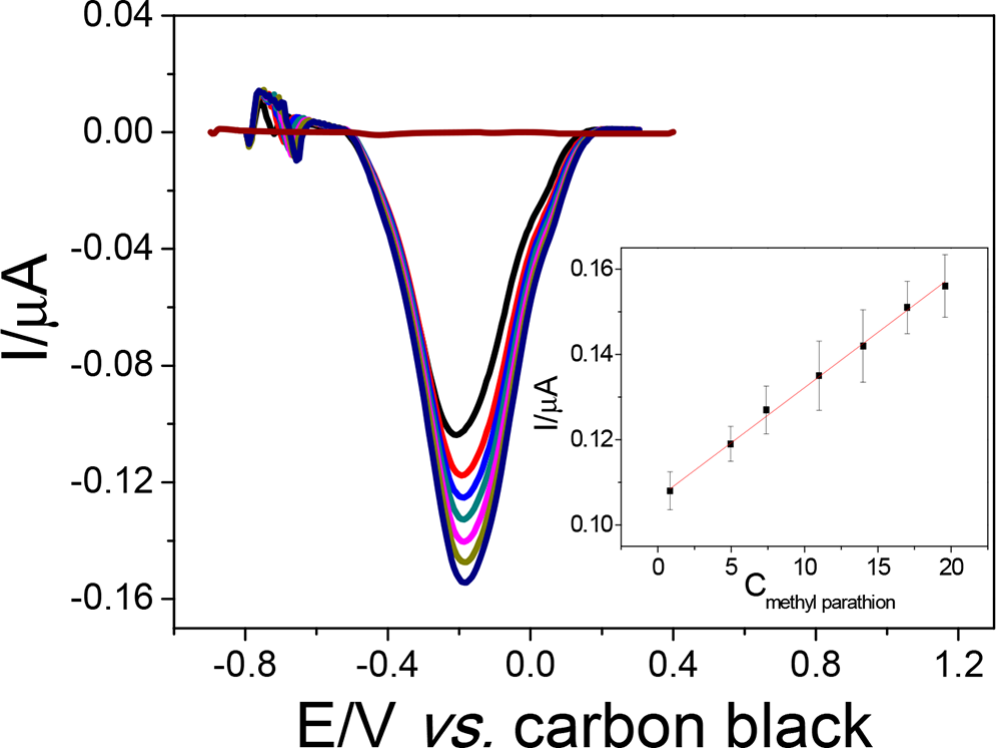
SWV voltammograms using the additive manufactured honeycomb system in the absence and presence of MP (0.85, 4.97, 7.40, 10.7, 14.0, 16.4, and 19.6 μmol L^–1^) in 0.1 mol L^–1^ phosphate buffer (pH 5.1). Conditions: step potential, 8.0 mV; square wave amplitude, 40 mV; frequency, 100 s^–1^; adsorption time, 420 s (*n* = 6).

Under the optimized parameters, a calibration curve was constructed by using the proposed honeycomb cell. Figure 6 shows the square wave voltammograms and the respective calibration curve. A linear behavior was observed for MP concentrations ranging from 0.85 to 19.6 μmol L^–1^, following the equation −*I* (μA) = 1.07(±0.01) + 0.026 (±0.002)*C*_MP_ (μmol L^–1^). The limit of detection (LOD) was 0.20 μmol L^–1^, obtained by the following relationship: 3 × blank standard deviation/slope of the calibration curve.

The proposed electrochemical system was then applied to detect MP in honey and tap water samples. For this purpose, the samples were spiked with known MP values, included in the calibration curve previously acquired. The obtained recovery results range from 97.8 to 112%, which are presented in Table 1. From these results, it is possible to conclude that the developed sensor presented a satisfactory analytical performance for the detection of MP in food (honey) and environmental (water) samples. Also, the reproducibility study was performed with six electrodes under the optimized values voltammetric values in the presence of 5.0 × 10^–5^ mol L^–1^ MP in 0.1 mol L^–1^ phosphate buffer medium (pH 5.1) and 420 s of adsorption time. As shown in Figure S5 (Supporting Information), the results provided the RSD of 4.8%, showing excellent values between the electrodes printed in the same electrochemical honeycomb cell. Moreover, the study of interferents was not carried out since the focus of this work is to demonstrate the versatility of the proposed system. To obtain high selectivity devices, the surface of the sensors should be prepared with some modifier, such as complexes, proteins, enzymes, antibodies, or aptamers.

**Table 1:** Recovery Values of MP Using the Additive Manufactured Honeycomb System with the Respective Standard Deviation Values (*n* = 6)

samples		added (μmol L^–1^)	measured (μmol L^–1^)	recovery (%)
honey	A	10.7	10.5 ± 1.2	97.8
	B	16.5	18.3 ± 0.5	112
tap water	C	7.40	7.30 ± 0.80	98.5
	D	10.0	10.1 ± 0.9	101

Upon inspection of the literature, it is possible to find several works involving the electrochemical detection of MP using sensors. In this sense, Table 2 presents some data from the works that propose the electrochemical detection of MP and the developed additive manufactured honeycomb system. It is worth noting that no studies were found involving the additive manufactured sensors for this analyte. In addition, we can observe that the compared works present similar analytical characteristics, such as linear ranges and LODs. It can also be noted that some of the published works require modified electrodes that are more complex, making them more labor-intensive than the proposed AM electrodes. Although the adsorption time is necessary, the analytical performance of the AM electrode makes its application attractive, with a reproducible system of 6 electrodes that can be used simultaneously at a relatively low cost. In addition, the system is simple to prepare and did not require postprocessing of the electrodes for its use.

**Table 2:** Comparison of the Analytical Characteristics of the Additive Manufactured Honeycomb System and Those Reported in the Literature for the Detection of MP^a^

sensor	LDR (μmol L^–1^)	LOD (μmol L^–1^)	ref
RGO/Pd-tetraphenylporphyrin	0.1–125	0.007	(42)
ZrO_2_	0.001–2.0	0.0005	(43)
CPME-AB	0.1–70	0.039	(44)
CP5-rGO/GCE	0.001–150	0.0003	(7)
additive manufactured CB/PLA	0.85–19.6	0.20	this work

a LDR, linear dynamic range; LOD, limit of detection; RGO/Pd-tetraphenylporphyrin, reduced graphene oxide/palladium tetraphenylporphyrin nanocomposite; ZrO_2_, ordered mesoporous zirconia; CPME-AB, carbon paste electrode modified with activated biochar; CP5-rGO/GCE, cationic water-soluble pillar[5]arene and reduced graphene nanocomposite.

To demonstrate the versatility of the proposed architecture, the same model was printed with central divisions to transform the proposed cell into 3 individually compartmentalized cells. For this purpose, the counter and reference electrodes were split into 3, giving a total of 3 distinct cells, each with 1CE, 1RE, and 2 WE, as observed Figure S6. This model can be applied to different analytes or even different concentrations of analyte at the same time.

Cyclic voltammetry measurements were performed on this new cell. Figure S7 shows the cyclic voltammogram obtained in the presence of 1.0 mmol L^–1^ [Ru(NH_3_)_6_]^3+/2+^ in 0.1 mol L^–1^ KCl using the honeycomb cell at a scan rate of 25 mV s^–1^. We can observe the well-defined anodic peak current with a value of 10 μA and the cathodic peak current of 16.8 μA, with the Δ*E*_p_ value of 100 mV. Using the 6 electrodes presented in the cell, it is possible to observe voltammograms with similar profiles and magnitudes (Figure S7), which provided the RSD of 4.3%. The electroactive areas of the working electrodes were estimated by cyclic voltammetry (Figure S5) (inset Figure S8) using the same conditions of the experiments presented previously. The average electroactive area obtained for the 6 electrodes was 0.004 cm^2^. The *k*^0^_obs_ was determined by the Nicholson method^40^ with the CV data obtained, which the obtained value was 2.81 ± 0.03 × 10^–3^ s^–1^. As the design and manufacture of the electrode portions of the cell remained unchanged from the noncompartmentalized honeycomb cell, the electroactive area and heterogeneous electron transfer constant of the compartmentalization proved identical. This further proves the great reproducibility of 3D-printed electrodes.

## 4 Conclusion

In this work, we propose a novel electrochemical cell with six working electrodes, auxiliary and reference electrodes, in a honeycomb-inspired device. This system did not require chemical/electrochemical treatment to carry out the electrochemical measurements. An IDEX printer was used to print nonconductive PLA and PLA/carbon black (conductive) filaments simultaneously, which demonstrated high-quality printing since no leakage was observed between the measurements performed. Therefore, the additive manufacturing process is fast and has a low production cost, within the accuracy and replicability of the technique, and the proposed device supports localized batch production. The device was also applied to the detection of MP, which presented a linear range from 0.85 to 19.6 μmol L^–1^, with a limit of detection of 0.20 μmol L^–1^, using square wave voltammetry. The 3D-printing process proved to be simple and relatively low cost, which was applied for the determination of MP in samples of tap water and honey. Also, the six working electrode arrays allowed the acquisition of a great number of measures in a short time with high precision. Therefore, this system is an interesting alternative for field use for the determination of MP in food and environmental samples.

## Supporting Information Available

Real images of the 3D-printed honeycomb system; contact angle measurements; EIS data; square-wave and cyclic voltammograms (PDF)
Video of the honeycomb being printed (MP4)
