# Supplementary material for: Novel Additive Manufactured Multielectrode Electrochemical Cell with Honeycomb Inspired Design for the Detection of Methyl Parathion in Honey Samples
Source: ACS Meas Sci Au. 2023 Apr 6;3(3):217–25. doi: 10.1021/acsmeasuresciau.3c00003 (PMC10288609; doi:10.1021/acsmeasuresciau.3c00003)
Supplement: Supplementary file 1 — tg3c00003_si_001.pdf [file tg3c00003_si_001.pdf]

Supplementary Material

Novel Additive Manufactured Multielectrode Electrochemical Cell with Honeycomb Inspired Design for the Detection of Methyl Parathion in Honey Samples

Bruno Campos Janegitz[a], Robert D. Crapnell[b], Paulo Roberto de Oliveira[a], Cristiane Kalinke[c], Matthew J. Whittingham[b], Alejandro Garcia-Miranda Ferrari[b], Craig E. Banks[b*]

[a]Department of Nature Sciences, Mathematics, and Education, Federal University of São Carlos, 13600-970, Araras, São Paulo, Brazil

[b]Faculty of Science and Engineering, Manchester Metropolitan University, Manchester M1 5GD, United Kingdom

[c]Institute of Chemistry, University of Campinas (Unicamp), 13083-859 Campinas, São Paulo, Brazil

*Corresponding authors: brunocj@ufscar.br; C.Banks@mmu.ac.uk

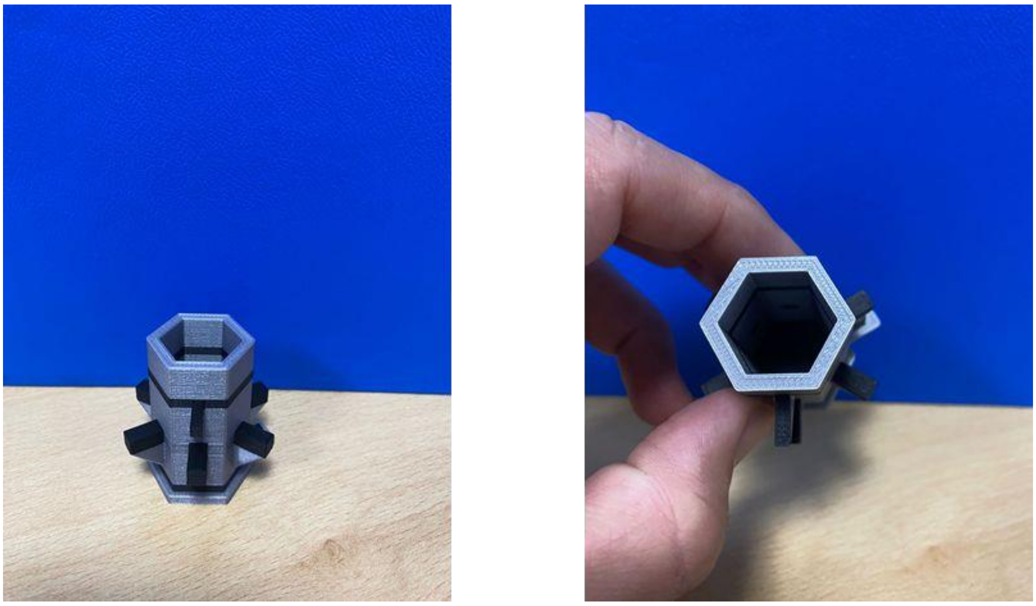

Fig. S1. Real images of the 3D printed honeycomb system, with 6 working, reference, and auxiliary electrodes.

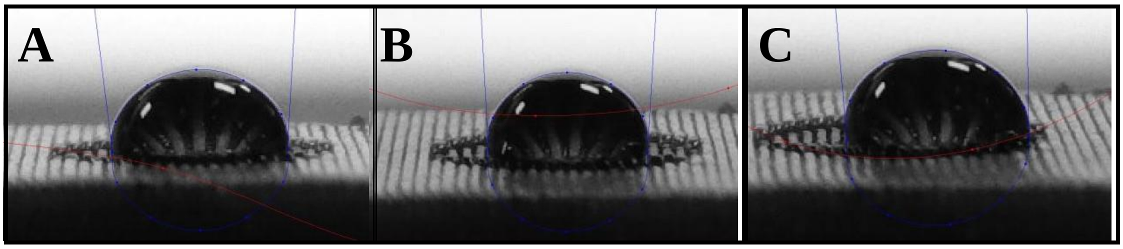

Fig. S2. Contact angle measurements performed for the conductive 3D printed electrode (carbon black/PLA filament based) using water as a solution (n = 3).

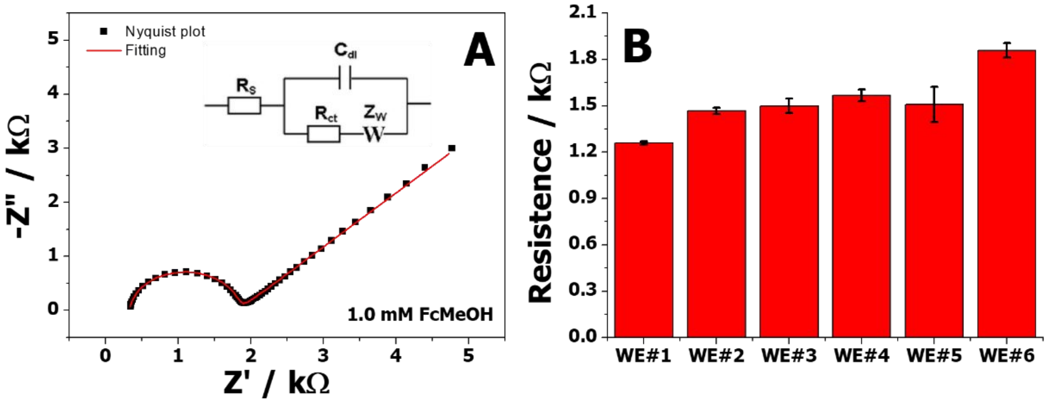

Fig. S3. (A) Representative Nyquist plot (■), and the fit (—) obtained from the equivalent Randles circuit (insert). (B) R_{ct} values obtained from the 6 WEs (n = 3).

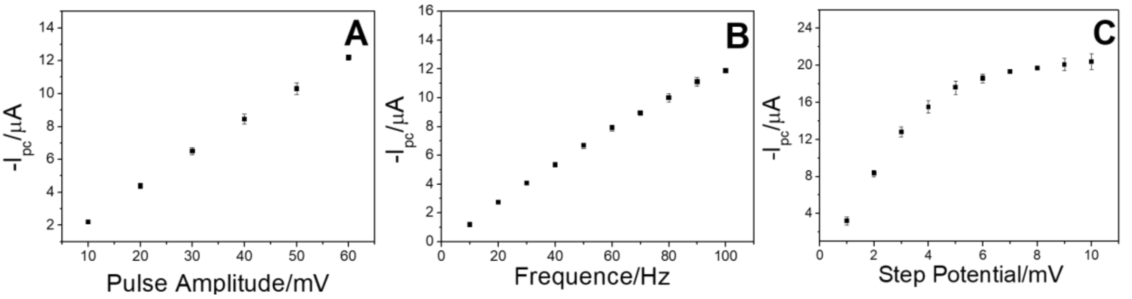

Fig. S4. Correlation results obtained between the intensity of the cathodic peak current ($-I_{pc}$) *vs.* (A) Pulse amplitude, (B) Frequence, and (C) Step potential for the detection of MP quantification by SWV.

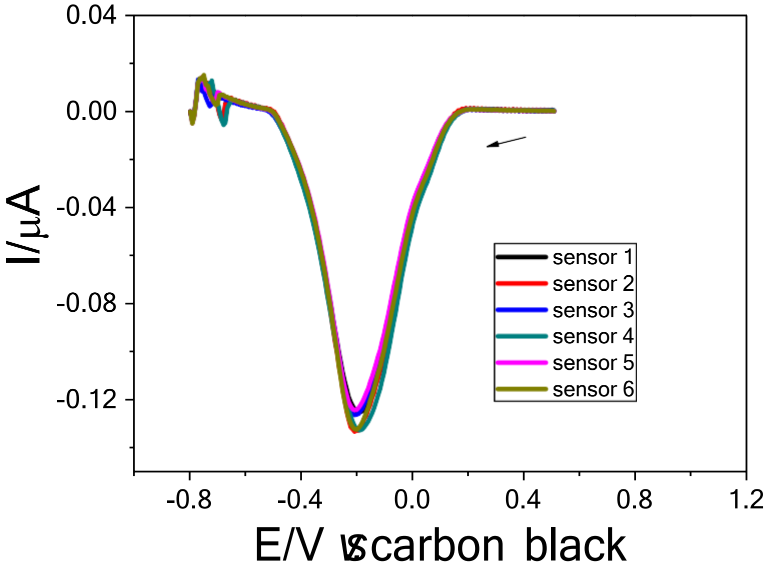

Fig. S5. Simultaneous SWV voltammograms using the 3D printed honeycomb system in the presence of 5.0×10^{-5} mol L^{-1} MP, in 0.1 mol L^{-1} phosphate buffer medium (pH 5.1). Conditions: step potential: 8.0 mV; square wave amplitude: 40 mV; frequency: 100 s^{-1}; adsorption time: 420 s.

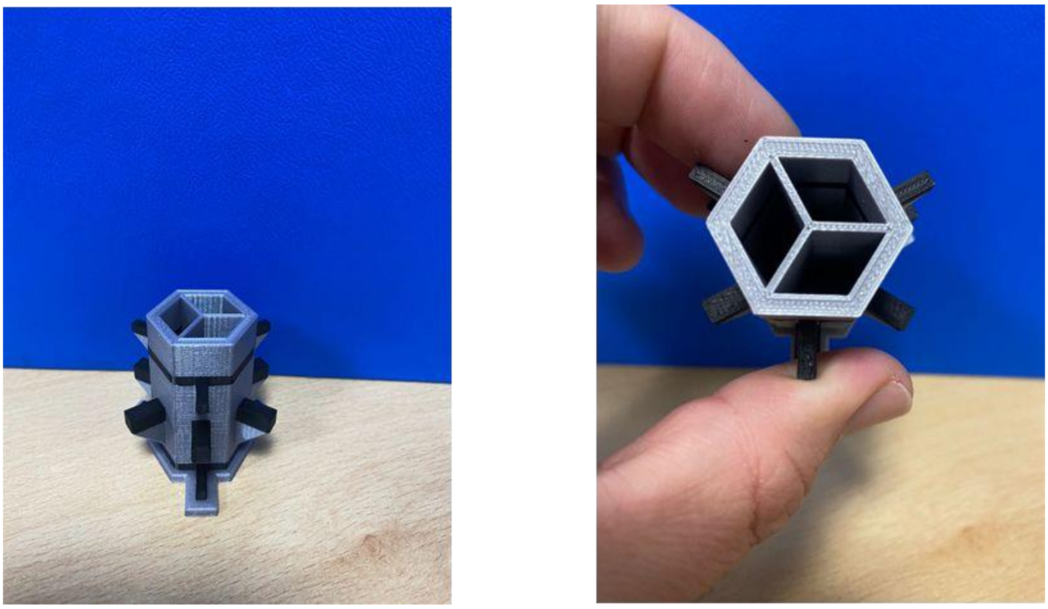

Fig. S6. Real images of the 3D printed honeycomb system (divided into 3 cells), with 6 working, reference, and auxiliary electrodes.

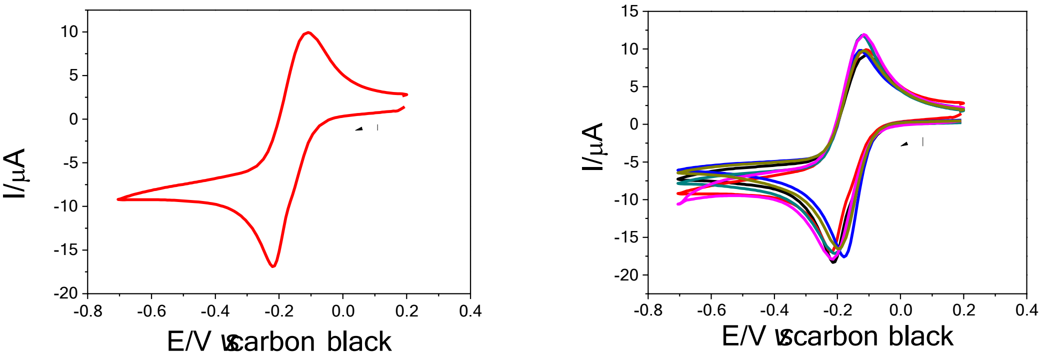

Fig.S7. Cyclic voltammograms of 3D printed honeycomb system obtained in the absence (black) in the presence (red) of 1.0 mmol L^{-1} $[Ru(NH_3)_6]^{3+/2+}$ in 0.1 mol L^{-1} KCl, at a scan rate of 25 mV s^{-1} using one electrode (A) and six electrodes (B).

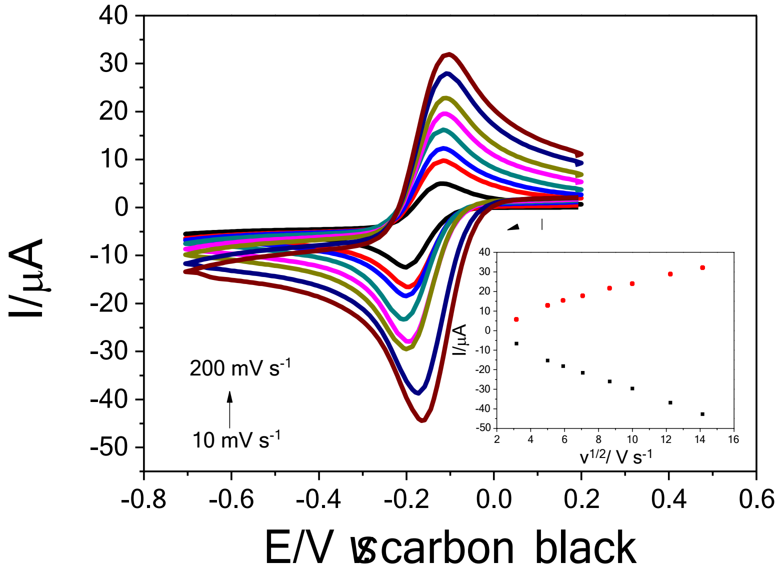

Fig. S8. Cyclic voltammograms of honeycomb system obtained in the presence of 1.0 mmol L^{-1} $[Ru(NH3)_6]^{3+/2+}$ in 0.1 mol L^{-1} KCl, at a scan rate of 10; 25; 35; 50; 75; 100; 150 and 200 mV s^{-1} (a) I x $v^{1/2}$ plot (inset).
